# Printing MEMS: Application of Inkjet Techniques to the Manufacturing of Inertial Accelerometers

**DOI:** 10.3390/mi14112082

**Published:** 2023-11-10

**Authors:** Roberto Bernasconi, Gabriele Pietro Invernizzi, Elisa Gallo Stampino, Riccardo Gotti, Davide Gatti, Luca Magagnin

**Affiliations:** 1 Dipartimento di Chimica, Materiali e Ingegneria Chimica “Giulio Natta”, Politecnico di Milano, Via L. Mancinelli 7, 20131 Milano, Italy; gabrielepietro.invernizzi@mail.polimi.it (G.P.I.); elisa.gallo@mail.polimi.it (E.G.S.); luca.magagnin@polimi.it (L.M.); 2 Dipartimento di Fisica, Politecnico di Milano e IFN-CNR, Via G. Previati 1/C, 23900 Lecco, Italy; riccardo.gotti@polimi.it (R.G.); davide1.gatti@polimi.it (D.G.)

**Keywords:** inkjet printing, SU-8, MEMS, accelerometer

## Abstract

In the last few years, the manufacturing of microelectromechanical systems (MEMS) by means of innovative tridimensional and bidimensional printing technologies has significantly catalyzed the attention of researchers. Inkjet material deposition, in particular, can become a key enabling technology for the production of polymer-based inertial sensors characterized by low cost, high manufacturing scalability and superior sensitivity. In this paper, a fully inkjet-printed polymeric accelerometer is proposed, and its manufacturing steps are described. The manufacturing challenges connected with the inkjet deposition of SU-8 as a structural material are identified and addressed, resulting in the production of a functional spring-mass sensor. A step-crosslinking process allows optimization of the final shape of the device and limits defects typical of inkjet printing. The resulting device is characterized from a morphological point of view, and its functionality is assessed in performing optical readout. The acceleration range of the optimized device is 0–0.7 g, its resolution is 2 × 10^−3^ g and its sensitivity is 6745 nm/g. In general, the work demonstrates the feasibility of polymeric accelerometer production via inkjet printing, and these characteristic parameters demonstrate their potential applicability in a broad range of uses requiring highly accurate acceleration measurements over small displacements.

## 1. Introduction

Micro-ElectroMechanical Systems (MEMS) are ubiquitous in a modern world dominated by information technology. Microfabricated actuators [1], sensors [2] and transducers [3], in general, are present in common household devices like smartphones, automobiles and electronic devices. Consequently, the MEMS market generates USD 12.1 billion per year in revenue globally [4] and drives a significant amount of applied research. MEMS are currently manufactured by state-of-the-art microfabrication techniques borrowed from the integrated circuits fabrication sector [1,3]. Such techniques, which include, for example, lithography and vapor phase deposition (PVD), are normally optimal for a large-scale production of identical devices. However, due to the high initial investment cost required, they are not advantageous for small-scale production of highly customized devices. Moreover, lithography is an intrinsically bidimensional technique. This is why production of highly customized MEMS characterized by a true tridimensionality is considerably challenging. Finally, conventional MEMS manufacturing is a rather complex process, characterized by a great number of subtractive and additive steps.

To overcome these limitations, some alternative approaches have been proposed. The use of additive manufacturing (AM), for example, can strongly limit the necessity of subtractive steps in the production of MEMS. AM-fabricated MEMS structures can potentially grow in the third dimension, allowing innovative geometries and functionalities [5]. Furthermore, AM techniques can be combined to yield hybrid production approaches that highlight the advantages of each technology [6]. The main limitations for most AM technologies are represented by the relatively large feature sizes obtainable and by the sometimes limited selection of usable materials [7]. Nevertheless, a variety of AM techniques has been successfully applied to the fabrication of MEMS [8,9,10]. The production of inertial sensors has been demonstrated using techniques like stereolithography (SLA) [11,12,13,14], fused deposition modelling (FDM) [15,16,17], multijet printing (MJP) [18] and two-photon lithography (TPL) [19].

Another interesting approach for MEMS production is inkjet printing (IJP). IJP is a material deposition technique based on the controlled emission of droplets from a nozzle. The droplets contain the material of interest in the form of particle suspension or in solution and are directed toward a substrate, on which they impact and form a layer [20]. This methodology has been employed as an advanced material deposition technique in many fields, including, for example, OLED production [21], advanced packaging [22], electrochemical [23,24] and chemiresistive [25,26,27] sensor manufacturing, battery development [28] and thin layer deposition for solar energy conversion [29] or photonic applications [30]. IJP has also been investigated as a supporting technique for MEMS manufacturing [31,32,33,34]. Indeed, IJP can be potentially used not only as a complementary technique but also to directly produce MEMS. Due to its high throughput, flexibility and simplicity, printing constitutes an attractive option for MEMS manufacturing [35]. Andò et al., for example, developed polymeric accelerometers by inkjet-printing sensing layers on contoured PET sheets [36,37,38,39].

In the present work, the fabrication of fully inkjet-printed uniaxial accelerometers is carried out for the first time. With respect to the existing literature, the body of the devices hereby presented is completely inkjet-fabricated. Inkjet-printed SU-8 was selected as constituent material for the devices. This photocurable polymer is characterized by a relatively stable viscoelastic behavior in a relatively wide temperature range [40,41]. In light of these properties, SU-8 has been already used in many studies to fabricate accelerometers [42,43,44]. Furthermore, polymeric accelerometers are characterized, in general, by high sensitivities [45,46] and ease of manufacturing [47]. Finally, the use of SU-8 offers the possibility to contain costs as compared to other jettable materials, like metallic nanoparticle suspensions. Using a jettable solution of SU-8, uniaxial accelerometers based on a prototypical design containing a seismic mass and two springs were successfully printed. The resulting devices were extensively characterized to individuate the peculiarities introduced by the inkjet manufacturing process. In particular, a dimensional, morphological and mechanical characterization was carried out. The accelerometers were also dynamically actuated to assess their functionality by means of piezoelectric actuation and optical readout. The SU-8 accelerometers described in the present work could find potential application as low-g and high-sensitivity devices [48] in the anti-seismic field or in high-precision remote navigation.

## 2. Materials and Methods

### 2.1. Design and Finite Element Analysis (FEA)

FEA was carried out using COMSOL Multiphysics version 5.6. Tetrahedral elements were employed to discretize the 3D model of the devices. A refined mesh, characterized by two elements in the out-of-plane thickness, was used on the two springs and in proximity of the two ends to guarantee good accuracy of the results. A coarse mesh, on the contrary, was generated on the proof mass and on the two anchors. The simulation was initially performed on the device presenting an ideal shape, and in this case, the mesh was constituted by 150,932 elements. After the realization and characterization of the device, a more refined simulation was performed, generating a mesh of 145,366 elements. Fixed constraint boundary conditions were applied on the surface of the anchors in contact with the substrate. Damping was modelled by imposing an isotropic structural loss factor (*η_s_*) equal to 0.015, which constitutes a reasonable value in air.

### 2.2. Materials

SU-8 2005 was purchased from Micro Resist Technology GmbH (Berlin, Germany) and was diluted using >99% pure cyclopentanone acquired from Sigma Aldrich (Milano, Italy). Si wafers covered by a layer of 100 nm of gold applied by sputtering were used as substrates.

### 2.3. Device Fabrication

An insulating aluminum-based adhesive tape, acquired from 3M (Pioltello, Italy), was used to mask the area for selective Zn deposition (adhesive material = acrylic adhesive, adhesion force = 6 N cm, working temperature = 40–160 °C, thickness = 0.09 mm). The Zn sacrificial layer was electrodeposited using the following solution: 0.5 M ZnCl_2_, 2.5 M NH_4_Cl, 1 g L^−1^ PEG 8000, and 1 g L^−1^ thiourea. pH was corrected to 6 with HCl or NH_4_OH, and the deposition was carried out under the following conditions: no stirring, 50 °C as temperature of the solution and 20 mA⸱cm^−2^ as current density. A mixed-oxide-coated Ti grid was used as the anode, and the current was applied for 32 min, 18 s. The structure of the accelerometer was created by inkjet-printing SU-8 with a Dimatix DMP printer manufactured by Fujifilm (Tokyo, Japan) following the methodology available in the literature [49]. Briefly, SU-8 2005 was diluted with cyclopentanone to yield a 40% wt. solution, which was loaded into a standard Dimatix DMP 10 pL cartridge. Inkjet printing was carried out using a 15 μm drop spacing, a voltage of 26 V, a print height equal to 0.7 mm, a jetting frequency of 1 kHz and 60 °C as the temperature for both the printing plate and the cartridge. The inkjet-printing SU-8 structure was cured following two distinct approaches: standard crosslinking or step crosslinking. The first was carried out at the end of the printing procedure, once the device was removed from the plate, and simply consisted of the standard procedure suggested by the SU-8 manufacturer. Curing times and temperatures depended on the thickness of the deposited SU-8 layer, and in the case here described, the following procedure was applied: soft bake at 65 °C for 4 min, UV exposition for 10 min and post-exposure bake at 95 °C for 5 min in order to guarantee the crosslinking of SU-8 and create the final structural layer. In the case of step crosslinking, the sample was left on the printing plate, and the crosslinking was carried out in situ following a technique previously described [49]. In detail, the printing process was started and then paused after 2 layers, heat was provided locally with a portable heater, and light was applied with a portable UV lamp. In this case, different crosslinking conditions were employed due to the lower thickness of the uncured layer: soft bake at 65 °C for 1 min, UV exposition for 1 min and post-exposure bake at 95 °C for 1 min. At the end of the crosslinking, the printing process was resumed, 2 more layers were printed, and the process was repeated until the desired thickness was reached. At the end of the SU-8 deposition step, the sacrificial Zn layer was dissolved using a 50 g L^−1^ citric acid solution in water. After this step, the devices were slowly and carefully extracted from the etching solution, washed via water immersion and finally left in air to naturally dry. Devices made with the standard reticulation crosslinking procedure were named ANS, while the devices manufactured with the step reticulation crosslinking approach were named AWS.

### 2.4. Devices’ Morphological and Mechanical Characterization

Optical microscopy (OM) images were acquired using a Leica DFC 290 stereomicroscope. For laser profilometry tests, a UBM Microfocus laser profilometer was employed. The scanning electron microscope (SEM) used in the present work was a Zeiss EVO 50 EP, combined with an Oxford Inca Energy EDS setup. Atomic force microscopy (AFM) was performed using a NT-MDT Solver Pro. SU-8 mechanical properties were characterized by means of Vickers microindentation using a Fischerscope 57 HCV indenter. The test was carried out on one of the two anchor points of the devices. A load equal to 5 mN and a loading time of 10 s were employed.

### 2.5. Device Testing

The functional characterization of the accelerometers was achieved with a Michelson interferometer, where the moving mirror was replaced by the proof mass. In order to increase its reflectivity, a few nm of gold was applied on the surface of the device by means of magnetron sputtering. The probe source was a linearly polarized helium-neon (He-Ne) laser (Melles Griot model 25 LGP 193) emitting a power of 5 mW at a wavelength of 543.5 nm and characterized by a beam diameter of 890 μm. The device under test was glued on a P-403-00 ring piezoelectric actuator (PZT), manufactured by Piezosystem Jena, using a nitrocellulose film. The PZT was driven by a waveform generator (Agilent 33220A) followed by an amplifier (FLC electronics A400) providing a fixed gain of 20. A silicon photodetector and two attenuators (Thorlabs NDL-10S-2) completed the setup. The resonance characterization was carried out providing a sinusoidal signal with a fixed amplitude voltage to the PZT and measuring the displacement as a function of the signal frequency. For the ring-down characterization, the accelerometer was driven at the resonance frequency (*f_res_*), and the decay constant of the signal (*τ*) was measured by averaging over hundreds of traces while periodically turning off the excitation signal. The quality factor (*Q*) was determined according to Equation (1).

(1) $$Q=\pi f_{res}\tau$$

The calibration curve for the displacement with respect to the variation of the impressed acceleration was obtained measuring the displacement as a function of the voltage provided to the piezoelectric actuator at a fixed frequency (measurements were made both in resonance and out of resonance to measure the baseline and in such a way to disentangle sample and PZT displacements). The acceleration was determined from the displacement of the accelerometer (Δ*x*) according to Equation (2).

(2) $$a={\left(2\pi f_{res}\right)}^{2}∆x$$

All the measurements were carried out both at low voltage (LV; 0–1 V) and high voltage (HV; 0–40 V), except the *τ* measurement, which was carried out at LV so that the interferometer was working in its linear zone. The HV measurements have a low noise-to-signal ratio but are more complex to process, while the LV measurements are acquired in the linear zone of the interferometer but are noisier and have the problem of drift of the working point related to the long-term drift of the Michelson interferometer arms (which is related to environmental conditions like temperature variations and vibrations).

### 2.6. Data Reproducibility

The reproducibility of the data was verified by testing multiple ANS and AWS. In detail, 3 ANS devices and 4 AWS devices were fabricated and tested.

## 3. Results and Discussion

### 3.1. Device Design

**Figure 1:**
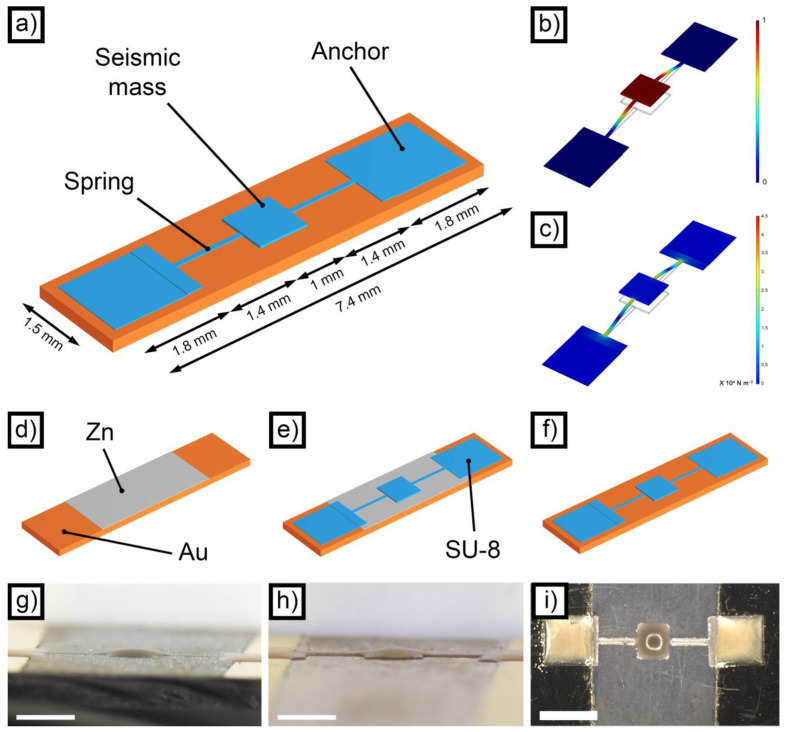
Scheme of the proposed accelerometer (**a**); FEM simulation of the first normal mode (**b**); FEM simulation of the internal stress distribution in a device (**c**); schematic representation of the different manufacturing steps: sacrificial layer deposition (**d**), SU-8 inkjet deposition (**e**) and sacrificial layer dissolution (**f**); OM side view of a device printed without step crosslinking before the dissolution of the sacrificial layer ((**g**) scale bar = 1 mm); OM side view of a device printed without step crosslinking after the dissolution of the sacrificial layer ((**h**) scale bar = 1 mm); OM top view of a device printed without step crosslinking after the dissolution of the sacrificial layer ((**i**) scale bar = 2 mm).

The inertial sensors described in the present work were designed following a simple spring-mass sensing principle (Figure 1a). In general, the accelerometer presents a moveable seismic mass capable of moving in the out-of-plane direction, which is sustained by two springs. When subjected to an acceleration, the mass exerts a force that moves it in a predictable and quantifiable way, allowing the indirect evaluation of the acceleration itself. From the mechanical point of view, the behavior of the device is described by Equation (3) [50].

(3) $$m\ddot{u}+b\dot{u}+ku=ma$$

where *u* represents the displacement of the proof mass, *b* is the damping coefficient, *k* is the elastic constant of the springs, *m* is the mass of the seismic mass and *a* is the external acceleration. It is worth noticing that Equation (3) contains, in addition to the inertial contribution coming from the mass and the elastic force exerted by the springs, a damping part resulting from the fluid in which the accelerometer is immersed or from the material that constitutes the springs.

The dimensions were selected following two main guidelines: the intrinsic limitations of the manufacturing technique employed and the frequency of the first normal mode of the accelerometer. Regarding the first point, inkjet printing is characterized by minimum feature sizes in the order of hundreds of micrometers. It was therefore not feasible designing the springs, which are the parts of the accelerometer characterized by the smallest dimensions, below a few hundreds of micrometers. In addition, due to the problems of surface tension and pattern broadening, typical of material jetting [49], the dimension of the mass and the anchor points were limited to the millimeter range. Regarding the second point, the frequency of the first normal mode was tuned to remain in the 1 kHz range, well below the resonance frequency of the PZT employed. At higher frequencies, the precision of the measurement could be negatively affected.

In light of these considerations, the dimensions reported in Figure 1a and Figure S1 were selected at the end of an FEM-assisted optimization of the first normal mode (based on the 3D model visible in Figure S2). One of the key points for a proper simulation of the mechanical behavior of the accelerometers was the choice of a realistic set of properties for the structural material. SU-8 mechanical features, in fact, strongly depend on the type of commercial product employed, on its thickness, curing conditions, moisture level and testing conditions. As a result, reported values of its elastic modulus range from around 5 GPa [51] to less than around 1.5 GPa [52]. Considering the thickness and the curing conditions of the SU-8 layer described in the present work, a realistic literature value of 2.2 GPa was employed [52,53].

The nominal thickness of the SU-8 deposited using inkjet printing was set to 20 μm, but the limited amount of material present in the mass resulted in a frequency equal to 901 Hz. To facilitate the testing, it was decided to decrease the frequency by increasing the amount of material deposited on the device. Since uniformly increasing the thickness of the whole device would have resulted in an excessive rigidity of the springs (and even higher frequencies), it was decided to enhance only the thickness of the mass by selectively inkjet-printing SU-8 only in correspondence with the mass itself. For this reason, 30 μm of additional material was printed on the mass, resulting in a local thickness of 50 μm. In this way, the expected frequency of the first normal mode (Figure 1b) was lowered to 645 Hz. The second and third normal modes were determined as well, and they are reported in Figures S3 and S4, respectively. Finally, the distribution of the stresses according to von Mises was evaluated at the resonant frequency (Figure 1c). It is evident that the maximum concentration of stress is present in the springs in correspondence with the connections with the anchor points and the seismic mass. These regions, in fact, are critical in determining the performance of the devices.

### 3.2. Production Process

**Figure 2:**
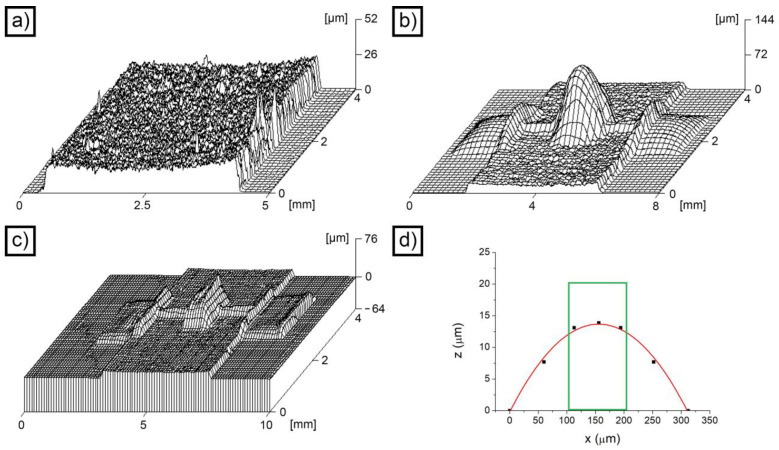
Height profile of the Zn sacrificial layer (**a**); height profile of an as-printed ANS device (**b**); height profile of an as-printed AWS device (**c**); theoretical (green) and experimental (red) profile of the SU-8 layer corresponding to a spring (**d**).

The production process employed to manufacture the accelerometers is schematized in Figure 1d–f. The first step (Figure 1d) was the deposition of the sacrificial layer of Zn, which was required to create the gap between the substrate and the seismic mass. In principle, it is possible to deposit the sacrificial layer by means of inkjet deposition, which could potentially result in a fully inkjet-based process. The reason for choosing electrodeposition, however, is related to the superior uniformity of the sacrificial layer obtained. Inkjet-printed layers, indeed, suffer from surface tension effects that tend to give rounded edges, as well as problems of localized thickness non-uniformity of the final layer. Figure S5, for example, shows the result obtained by jetting a layer of polyacrylic acid (PAA) from a solution containing 5% wt. PAA, 30% ethylene glycol and 65% wt. water. The uniformity of the layer appears acceptable, but some localized cavities can be observed. Moreover, the edges appear considerably rounded. In the case of electrodeposited Zn, on the other hand, the uniformity of the final sacrificial layer appears excellent, with no evident depressions (Figure 2a). The thickness of the layer, in this case, was 20.6 ± 2.3 μm.

Following Zn deposition, SU-8 was patterned via inkjet printing to form the structure of the accelerometer (Figure 1e). The polymer was printed on both the deposited Zn and the substrate, thus forming a freestanding structure (the mass-spring system) and two contact points connected to the substrate and capable of sustaining the suspended part. The result obtained at the end of the SU-8 deposition step can be observed in Figure 1g. Figure S6 depicts the monochromatic bitmap employed to print the structural material, which consisted of 10 printed layers of SU-8. Due to the necessity of locally increasing the thickness of the seismic mass, another bitmap (Figure S7) was used to deposit 15 additional layers of SU-8 only on its surface. The SU-8 layers were then cured; the samples made with the standard crosslinking procedure were named ANS, while the devices manufactured with the step crosslinking approach were named AWS.

As a final step, after curing the SU-8 layer, Zn was dissolved, and the freestanding structure was released (Figure 1f). The air gap that formed as a consequence of the dissolution process is clearly visible in Figure 1h and Figure S8, where a finished device is represented. Its height was measured from the OM images and was equal to 22 ± 3 nm. The same device has been photographed from the top, and the picture obtained is presented as Figure 1i. By looking at Figure 1h,i, the presence of a grey surface under the springs and the mass can be observed. This is the zone where the Zn layer used to be, and it is a direct consequence of the room temperature interdiffusion between Zn and the gold-coated substrate. The diffusion coefficient for Zn in Au, indeed, is considerably high, even at room temperature [54], leading to the formation of a layer containing AuZn_3_ and AuZn. These compounds are considerably more corrosion resistant than pure zinc, and they did not dissolve significantly during Zn removal. Their presence is irrelevant for the functioning of the device, and they do not introduce any significant issue for the manufacturing process.

### 3.3. Dimensional Analysis

As already mentioned, inkjet material deposition presents important challenges that reflect on the quality of the SU-8 layer and on the dimensional adherence of the final device to the designed 3D model. These problems are essentially related to the necessity to work with the material of interest in the form of a fluid, i.e., the ink jetted. In particular, the broadening effect [49] increases the nominal dimensions of the features that constitute the device, spreading the ink on a surface larger than the expected one. As a side effect, it also decreases the thickness of the deposited layers in some regions. The already cited surface tension effect, in turn, rounds the features printed, making impossible the creation of sharp edges and perfectly square sections [49].

The finished devices were analyzed from the dimensional point of view, evaluating their linear dimensions and their tridimensional profile. Table 1 reports the results obtained from the direct measurement of the dimensions of the ANS and AWS devices. These are compared with the expected values.

The most immediate observation considering the data reported in Table 1 is that, due to inkjet pattern broadening, external dimensions increased with respect to their theoretical values. The percentage variation observed can be correlated with the dimension of the feature considered, with larger features presenting increases of smaller percentages and smaller ones (like *d*) presenting large variations of more than 100%. Internal dimensions (like *e*), in turn, presented decreased values as compared to their theoretical values. Another important aspect that can be highlighted is the difference observed between the ANS and the AWS samples. In general, the deviation from the expected values was lower in the case of the AWS devices. This effect is a consequence of the step-crosslinking process, since the continuous crosslinking introduced by the step curing methodology avoids the periodic partial redissolution of the SU-8 layer occurring when the subsequent layer is printed on the previous one. Such partial redissolution, occurring in the ANS devices, allows fluid flow and material rearrangement on the surface, which boost pattern broadening.

For the tridimensional profile of the devices, the analysis allowed determination of the distribution of the material in the structure. Obviously, due to surface tension and capillarity effects, it was impossible to obtain a uniform 20 μm layer (50 μm on the seismic mass). In fact, by looking at the 3D profile of the ANS device shown in Figure 2b, a strong non-uniformity in the distribution of SU-8 can be clearly observed. In general, the material tends to accumulate in correspondence with the largest features (the seismic mass and the anchors) and to be partially sucked from the springs. The profiles appear rounded and characterized by sloping edges. In the case of the AWS devices, the situation appears radically different (Figure 2c). The edges look sharper, and the distribution of the material is more uniform. To give a comparison, the highest point visible in the 3D profile of the ANS device (on the seismic mass) was located at 144 μm, while the highest point for the AWS device was at 76 μm (always on the seismic mass). If the thickness of the Zn layer is removed, these two values translate into a maximum height of the seismic mass of 123 μm for the ANS device (+146% with respect to the expected 50 μm value) and 55 μm for the AWS device (+10% with respect to the expected 50 μm value). By comparing these two values, it appears evident that SU-8 was more evenly distributed on the AWS device, which demonstrated dimensional conformance to the nominal 3D model of the accelerometer.

The most crucial parts of the accelerometer, from the functional point of view, are the springs. They are the features that store mechanical energy during the movement of the mass, and the greatest stress accumulation can be observed in correspondence with their extremities (Figure 1c). It is therefore fundamental to evaluate their shape and compare it with the theoretical one. To carry out this task, the 3D profiles reported in Figure 2 were reprocessed, and the line profile in correspondence with the cross-section of a spring was plotted (Figure 2d). Due to the pattern spreading effect, the width of the spring (in red in Figure 2d) appeared considerably larger than the theoretical one (in green in Figure 2d). Moreover, the height of the spring was considerably reduced. In general, the numerical area of the section was comparable (1910 μm^2^ in place of 2000 μm^2^), but the distribution of the material changed considerably. The springs of the AWS device were characterized by a comparable morphology.

### 3.4. Morphological and Mechanical Characterization

**Figure 3:**
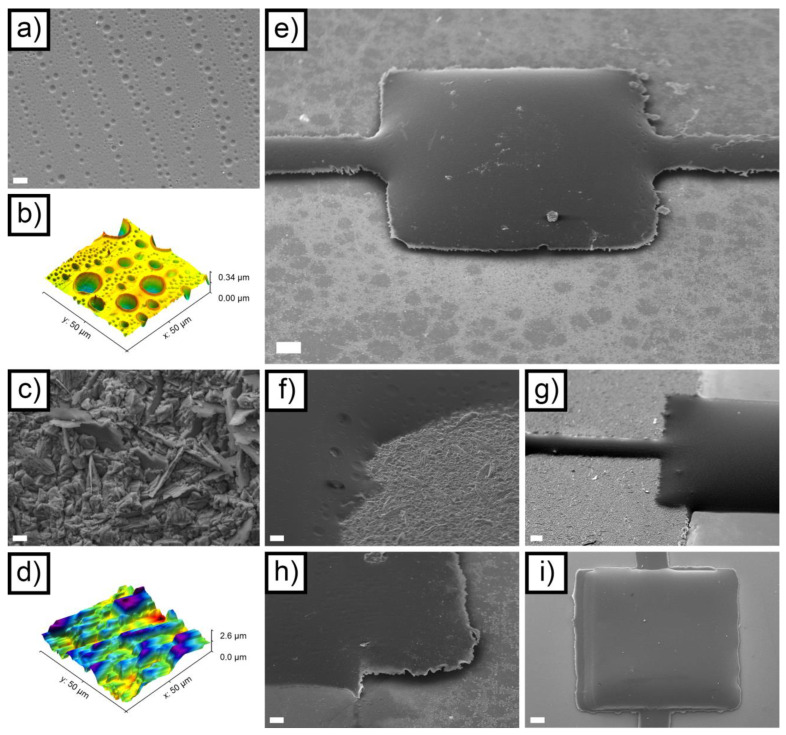
SEM image of the SU-8 layer ((**a**) scale bar = 6 μm); AFM image of the SU-8 layer (**b**); SEM image of the sacrificial Zn layer ((**c**) scale bar = 4 μm); AFM image of the sacrificial Zn layer (**d**); SEM image of an ANS device at the end of the manufacturing process ((**e**) scale bar = 100 μm); high magnification SEM image of an ANS device before removal of the Zn layer ((**f**) scale bar = 20 μm); SEM image of an ANS device before removal of the Zn layer ((**g**) scale bar = 100 μm); high magnification SEM image of an ANS device after removal of the Zn layer ((**h**) scale bar = 40 μm); SEM image of an AWS device after removal of the Zn layer ((**i**) scale bar = 100 μm).

At the end of the macroscopic dimensional analysis, the devices were analyzed to assess their morphology at the microscale. Figure 3a depicts the morphology of the SU-8 layer that constitutes the structure of the accelerometers. The most evident features present on the surface of the material are micrometric porosities organized in parallel lines. These are a result of the inkjet printing process, which deposits lines of droplets made of diluted SU-8. When the solvent evaporates, it leaves small cavities on the surface of the deposited layer, which are organized in lines separated by a distance that corresponds exactly to the drop spacing set on the Dimatix printer (15 μm). The crater-like morphology of these cavities can be better observed using AFM (Figure 3b). Figure 3 also highlights that the zones between the largest porosities, which are a few μm wide, are dotted by nanometric porosities that are almost invisible in the SEM. In general, despite these hierarchically organized porosities, the roughness of the SU-8 layer was low, presenting a value of R_a_ equal to 30.8 ± 2.7 nm for the 50 μm × 50 μm scanning area depicted in Figure 3b.

The Zn layer, which resulted from electrodeposition, was characterized by a considerably coarser morphology. The SEM image reported in Figure 3c shows a polyhedral structure, resulting from the crystalline growth typical of electrodeposited Zn. In addition to this, some lamellar structures can be observed in the same image. Figure 3d shows the AFM of the Zn layer, from which the roughness of the surface was calculated: 298.9 ± 8.2 nm for the 50 μm × 50 μm scanning area depicted in Figure 3b. It is interesting to point out that this is the same level of roughness that could be found on the bottom face of the springs and of the seismic mass after the dissolution of the sacrificial layer, since the morphology of the electrodeposited layer directly transferred to the SU-8 layer.

Figure 3e depicts the SEM morphology of the central section of the mass-springs system in an ANS device, after the removal of the Zn layer. The air gap under the system itself is clearly visible, and its good uniformity can be appreciated. As a result of the inkjet-induced effects, the corners present a characteristic rounded shape. Figure S9 shows a magnification of one of the two springs, evidencing the porosities present on the surface of the SU-8. Under the spring, the morphology of the interdiffused Zn-Au layer can be observed as well. This is characterized by a spotted morphology resulting from the formation of the intermetallics previously cited. Figure 3f,g shows the situation before the removal of the sacrificial Zn layer from an ANS device. The region where the springs connect to the anchor point (Figure 3f), similarly to the corners of the seismic mass, presented a rounded morphology. The bulge of the SU-8 layer, resulting from the surface tension–mediated effects, can be observed in Figure 3f (as well as in Figure 3e,g and Figure S9). Figure 3h shows the SEM morphology of the anchor point of an ANS device, corresponding to its suspended part. Even after the Zn dissolution process, the interface between Au and SU-8 appears stable and continuous, with no apparent cracks and delaminations. Figure 3i shows the SEM morphology of the seismic mass of an AWS device. The better uniformity of the SU-9 structural part, if compared to Figure 3e, can be plainly appreciated. In addition, some slight misalignment between the initial 10 printed layers and the subsequent 15 layers (printed only on the mass) can be observed. This peculiarity results from the need to realign the origin point for the printing when switching from Figure S6 to Figure S7.

As previously mentioned, SU-8 mechanical properties vary considerably according to many variables. Considering this intrinsic variability, the elastic modulus of the SU-8 layer deposited in the present work was assessed by means of microindentation. It was 1.6 ± 0.13 GPa for the ANS device and 2.1 ± 0.12 GPa for the AWS device. This marked difference is a result of the step-crosslinking process, which allowed increase of the crosslinking degree of the SU-8 and consequently improvement of the elastic modulus. The low elastic modulus observed without step curing was indicative of a low reticulation grade, with the possible presence of only partially cured regions inside the SU-8 layer.

### 3.5. Accelerometer Testing

**Figure 4:**
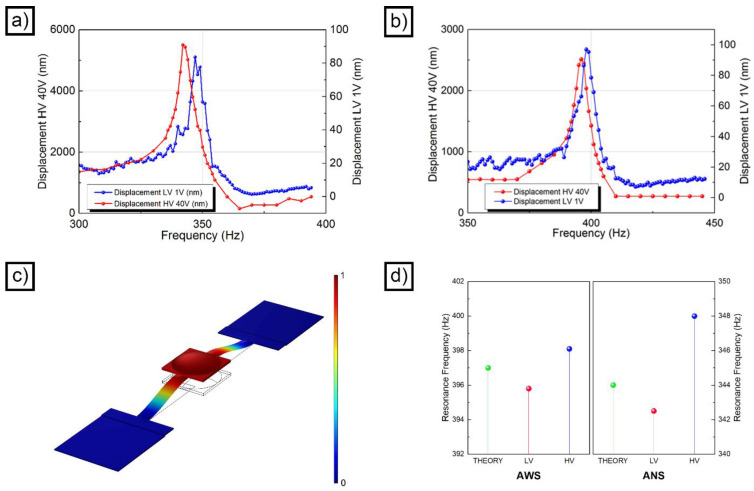
Resonance behavior of an ANS device (**a**); resonance behavior of an AWS device (**b**); FEM simulation of a device characterized by a realistic morphology (**c**); comparison between the resonance frequencies of the AWS and ANS devices with their theoretically predicted FEM values (**d**).

The functionality of the inkjet-printed polymeric accelerometers was investigated using the optical readout approach (Figure S10). As a first functional test, the resonance behavior of the devices was assessed. Figure 4a,b show the resonance curve obtained in LV (blue line) and in HV (red line) for the ANS and the AWS accelerometers, respectively. In both cases, the LV and HV curves have a very similar profile, with a full width at half maximum (FWHM) of about 9 Hz (AWS) and 8 Hz (ANS). In both accelerometers, there was a frequency shift between LV and HV, which was reproducible by repeating the measurement. Since it is independent of the accelerometer, we exclude that it may in any way be linked to the measuring system. The estimated resonance frequency for the AWS device was 395.8 Hz in the LV case and 398.1 Hz in the HV case, with a difference of 2.3 Hz between the two curves. In the case of the ANS accelerometer, the resonance frequency was 342.5 Hz in the LV case and 348 Hz in the HV case, with a difference of 5.5 Hz between the two curves.

The difference observed between the ideal device (Figure 1b) and the ANS device can be, in part, attributed to the geometrical alterations induced by the inkjet printing process. The springs, for example, were characterized by a non-ideal section, in which the distribution of the mass was different with respect to the designed one. This difference induces a variation of the moment of inertia of the section (*I_section_*). If the system is approximated to a doubly fixed beam, this variation influences the normal frequency (*f_res_*) of the device (Equation (4)).

(4) $$f_{res}\propto I_{section}\propto \iint_{A}^{}\rho dA$$

where *dA* represents an infinitesimal area, and *ρ* is the perpendicular distance of said area from an arbitrary axis. In addition to these geometrical considerations, the real elastic modulus of the structure was lower than the one used for the simulation. Finally, the other parts of the accelerometer, and especially the seismic mass, presented a non-ideal shape. The difference in resonance frequency observed between the AWS and the ANS samples is due to the step-crosslinking process. Indeed, step curing improved the mechanical properties of the material by enhancing the SU-8 reticulation degree.

Taking into account these considerations, the FEM simulation of the frequency response of the devices was refined and improved. First, the springs were 3D modelled using the real section, acquired from 3D profilometry (Figure 2d). Second, the experimental values for the elastic modulus of the SU-8 were implemented in the analysis. Finally, the shape of the seismic mass was modelled, in the case of the ANS device, to resemble the profile visible in Figure 2b. All these improvements led to the definition of a new 3D model (Figure S11), which yielded two realistic resonance frequencies: 404 Hz in the case of the AWS accelerometer (Figure 4c) and 352 Hz in the case of the ANS device. These two values reasonably match the two measured from interferometry (Figure 4d) and take in consideration the non-idealities introduced by the inkjet manufacturing process.

**Figure 5:**
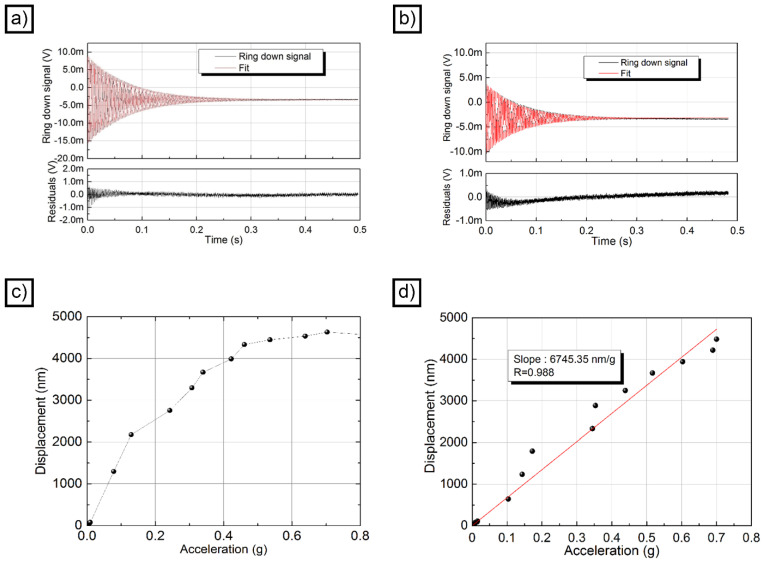
Ring-down experiment for an AWS device (**a**); ring-down experiment for an ANS device (**b**); calibration curve for an ANS device (**c**); calibration curve for an AWS device; linear fit is displayed as a red straight line (**d**).

The quality factor of the accelerometer was evaluated from the ring-down measurement, providing a sinusoidal excitation at the resonance frequency (397 Hz for AWS and 344 Hz for ANS). Once the oscillation reached a stationary condition, the signal was interrupted, and the decay constant τ was measured. The average of 50 acquisitions is represented (together with the fit for both accelerometers) in Figure 5a (AWS) and Figure 5b (ANS). The fit performed using an exponentially enveloped sinusoidal function allowed us to estimate a ring-down time of about 73 ms for AWS and 68 ms for ANS. Despite the drift of the interferometer arms due to environmental conditions, the residuals were quite low. The working point variation over time, clearly visible in the residuals, is responsible for the asymmetry of the profile. The uncertainty in the constant of decay was estimated at 4–5 ms, and a quality factor Q of around 91 ms for AWS and 73 ms for ANS could be assessed. The measurements clearly demonstrate that the superior mechanical properties of the AWS device translated into less damping and a higher quality factor.

Figure 5c represents the displacement of the single-accelerometer ANS to various accelerations (in both HV and LV), while Figure S12 shows only the points measured under LV conditions. It is evident, when looking at the figure, that the behavior of the accelerometer was non-linear, with a clear saturation at high levels of acceleration. A linear fit was carried out by taking two different subsets of points. In one case, the LV points and the first two HV points (those with acceleration less than 0.15 g) were selected. The linear fit obtained with the intercept fixed to 0 is represented by the red line in Figure S12. In the second case, all the LV points and the first 5 HV points (those with acceleration less than 0.4 g) were used. The linear fit obtained with the intercept fixed at 0 is represented by the green line in Figure S12. As can be seen, the slopes are very different in the two cases (16,566 nm/g for the first case and 11,352 nm/g for the second). What is more significant, however, is that the slope in both cases is very different from the trend exhibited by the LV points alone, whose fit is represented by the orange line in Figure S13 and has a slope of 8715 nm/g. This value is more comparable to the one obtained with the accelerometer AWS.

Figure 5d represents the displacement of the single-accelerometer AWS to various accelerations (in both HV and LV), while Figure S14 represents only the points measured under LV conditions. In this case, the response of the AWS sample was linear, and the saturation that is present in the ANS sample could not be observed. A slope of 6745 nm/g for the AWS sample was retrieved from a linear fit of the overall data imposing the zero intercept. Linear fit of the low voltage points (reported in Figure S14) confirms that the slope does not change in different acceleration regimes.

## 4. Conclusions

In the present work, SU-8–based accelerometers have been successfully manufactured employing material inkjet deposition. A sacrificial layer of zinc was electrodeposited to create the gap between the substrate and the moveable mass, while SU-8 was printed on a layer as a structural material of the final device. The experimentation evidenced specific challenges, typically observed in the case of inkjet-printed layers. In particular, the shape of the device was found to be significantly altered by surface tension effects taking place before the complete evaporation of the solvent from the SU-8 layer. Patterns were also found to be altered by the broadening effect, which is once again connected to the fluidity of the material prior to solvent evaporation. Step crosslinking was identified as a potential way to improve the morphology of the device and the mechanical properties of its structural material. Indeed, it was found to make the final shape more adherent to the theoretical one and to increase the elastic modulus of SU-8 by 31.3%. After dissolution of the sacrificial layer, the mass-springs system present in the accelerometers was released and allowed to freely move upon application of an external acceleration. Consequently, the performance of the devices was assessed by means of an optical readout. The application of step crosslinking induced evident effects on the properties of the accelerometers. The frequency of the first normal mode, for example, increased by 14.4–15.6% (HV or LV) when comparing devices obtained from step crosslinking with accelerometers obtained without. Step crosslinking also positively influenced the Q factor and the linearity of the finished device. Step-reticulated accelerometers presented the following relevant parameters: 0–0.7 g as the linear range, 2 × 10^−3^ g as the resolution and 6745 nm/g as the sensitivity. These values are in line with applications requiring highly accurate acceleration measurements over small displacements, like anti-seismic monitoring and biomedical sensing. To this extent, it will be fundamental to integrate a portable readout system into manufactured accelerometers. The most promising approach, from this point of view, is the deposition of a conductive metallic layer on the surface of the devices, enabling a capacitive readout of the mass displacement. Nevertheless, the results hereby obtained support the potential applicability of material jetting in the manufacturing of inertial MEMS.

## Figures and Tables

**Table 1:** Comparison between expected and measured dimensions for ANS and AWS devices. The dimensions reported correspond to those visible in Figure S1.

		ANS		AWS	
Dimension	Expected Value (μm)	Measured Value (μm)	Variation (%)	Measured Value (μm)	Variation (%)
*a*	1500	1614 ± 7	7.6	1541 ± 6	2.8
*b*	1500	1652 ± 9	10.1	1604 ± 7	7
*c*	300	226 ± 8	−24.6	271 ± 6	−9.8
*d*	100	231 ± 6	130.7	220 ± 6	120
*e*	1400	1294 ± 19	−7.5	1325 ± 8	−5.3
*f*	1000	1137 ± 7	13.7	1018 ± 13	1.8
*g*	1000	1172 ± 5	17.2	1157 ± 7	15.7

## Data Availability

The data that support the findings of this study are available from the corresponding author upon reasonable request.

## Supplementary Materials

The following supporting information can be downloaded at: https://www.mdpi.com/article/10.3390/mi14112082/s1, Figure S1. Scheme of the accelerometer. Figure S2. 3D model of the accelerometer. Figure S3. Second normal mode. Figure S4. Third normal mode. Figure S5. 3D profile of an inkjet printed PAA sacrificial layer. Figure S6. Bitmap used to print the accelerometer. Figure S7. Bitmap used to increase the weight of the seismic mass. Figure S8. OM image of the air gap present at the end of the manufacturing process in a ANS device. Figure S9. SEM image of one of the two springs in a ANS device. Figure S10. Modified Michelson interferometer. Figure S11. Realistic 3D model of a printed accelerometer. Figure S12. Displacement of the accelerometer ANS to various accelerations in LV regime. Figure S13. Fitting of the displacement vs. acceleration behavior in LV regime. Figure S14. Displacement of the accelerometer AWS to various accelerations in LV regime. Table S1. Dimensions of the accelerometer.
