# Supplementary material for: Printing MEMS: Application of Inkjet Techniques to the Manufacturing of Inertial Accelerometers
Source: Micromachines (Basel). 2023 Nov 10;14(11):2082. doi: 10.3390/mi14112082 (PMC10672808; doi:10.3390/mi14112082)
Supplement: Supplementary file 1 [file micromachines-14-02082-s001.zip › micromachines-2701245-supplementary.pdf]

<u>Supplementary material for:</u>

Printing MEMS: application of inkjet techniques to the manufacturing of inertial accelerometers

Roberto Bernasconi[1], Gabriele Pietro Invernizzi[1], Elisa Gallo Stampino[1], Riccardo Gotti[2], Davide Gatti[2], Luca Magagnin[1]*

1 Dipartimento di Chimica, Materiali e Ingegneria Chimica "Giulio Natta", Politecnico di Milano, Via L. Mancinelli 7, 20131, Milano (Italy)

2 Dipartimento di Fisica, Politecnico di Milano, Via G. Previati 1/C, 23900, Lecco (Italy)

* Corresponding author: roberto.bernasconi@polimi.it

Figure S1 schematizes the dimensions of the accelerometer. Such dimensions are detailed in table S1. Figure S2 shows the tridimensional model of the accelerometer employed for the FEM analysis.

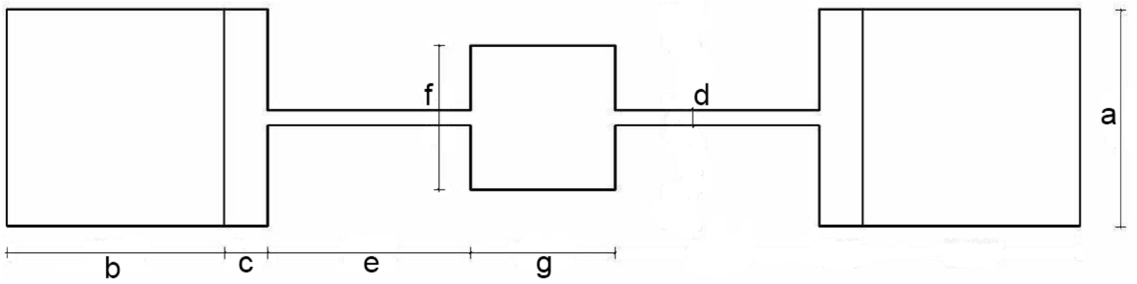

Figure S1. Scheme of the accelerometer

Table S1. Dimensions of the accelerometer

Dimension	Value (μm)
**a**	1500
**b**	1500
**c**	300
**d**	100
**e**	1400
**f**	1000
**g**	1000

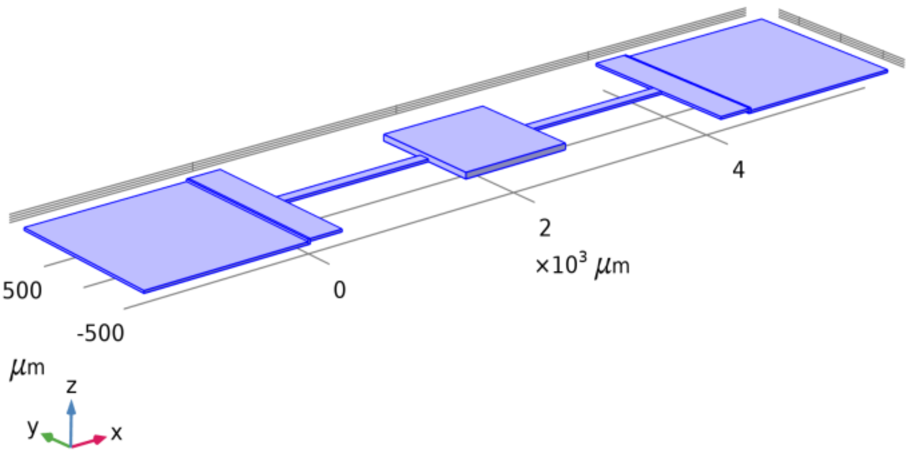

Figure S2. 3D model of the accelerometer

Figures S3 and S4 depicts the FEM simulation of the second and third normal modes, respectively.

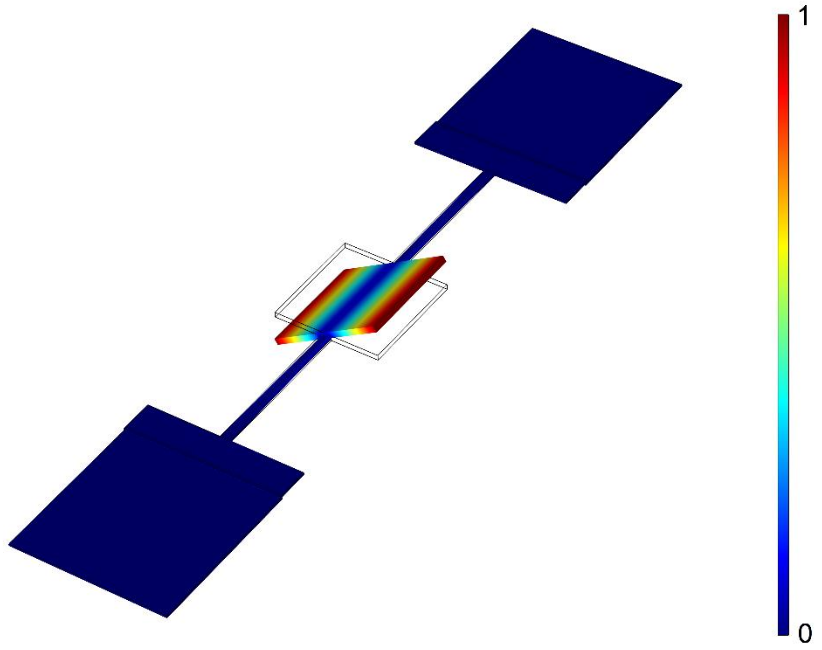

Figure S3. Second normal mode

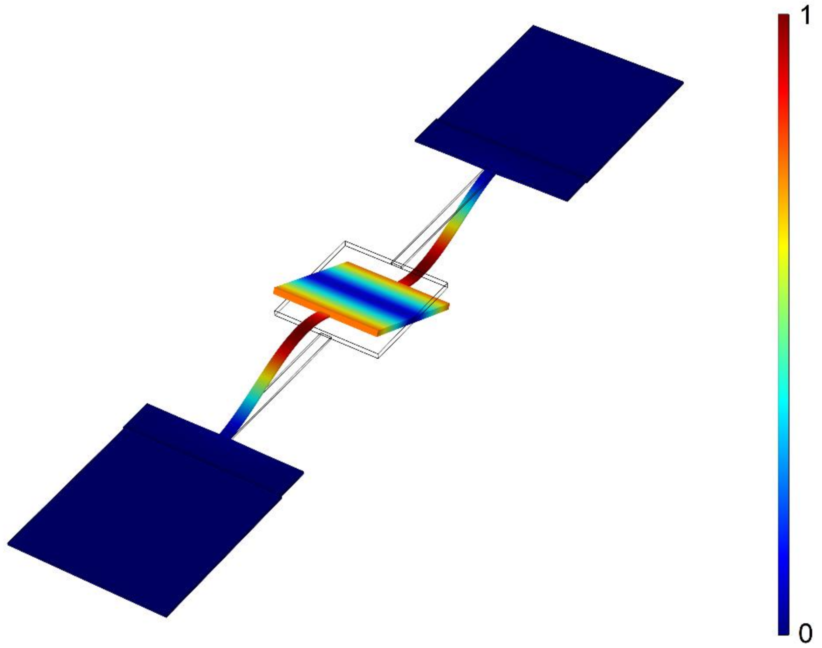

Figure S4. Third normal mode

Figure S5 shows the 3D profilometry of a sacrificial layer obtained jetting a solution containing 5 % wt. poly acrylic acid (PAA).

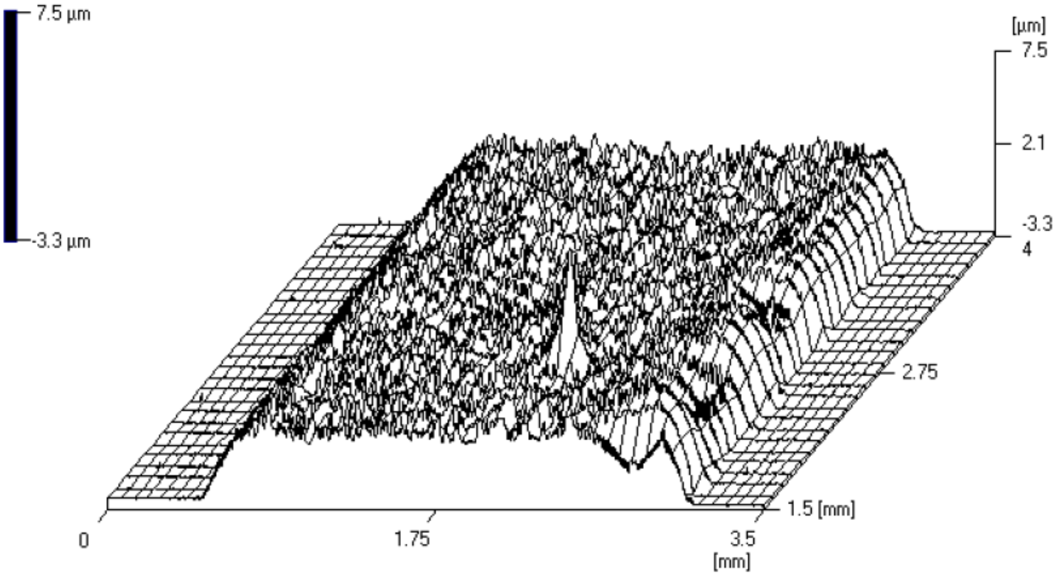

Figure S5. 3D profile of an inkjet printed PAA sacrificial layer

Figures S6 and S7 depict the bitmaps employed to print the structure of the accelerometer (figure S6) and to increase the thickness of the seismic mass (figure S7).

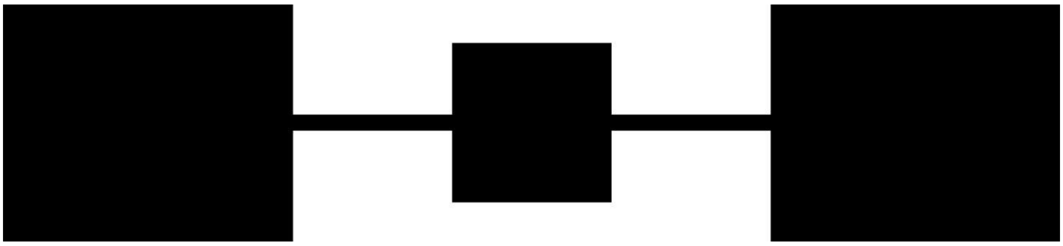

Figure S6. Bitmap used to print the accelerometer

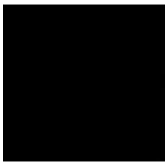

Figure S7. Bitmap used to increase the weight of the seismic mass

Figure S8 is an optical microscope (OM) image of the air gap present between the structure of a ANS accelerometer and the substrate. Figure S9 shows the SEM morphology of a spring of a ANS device.

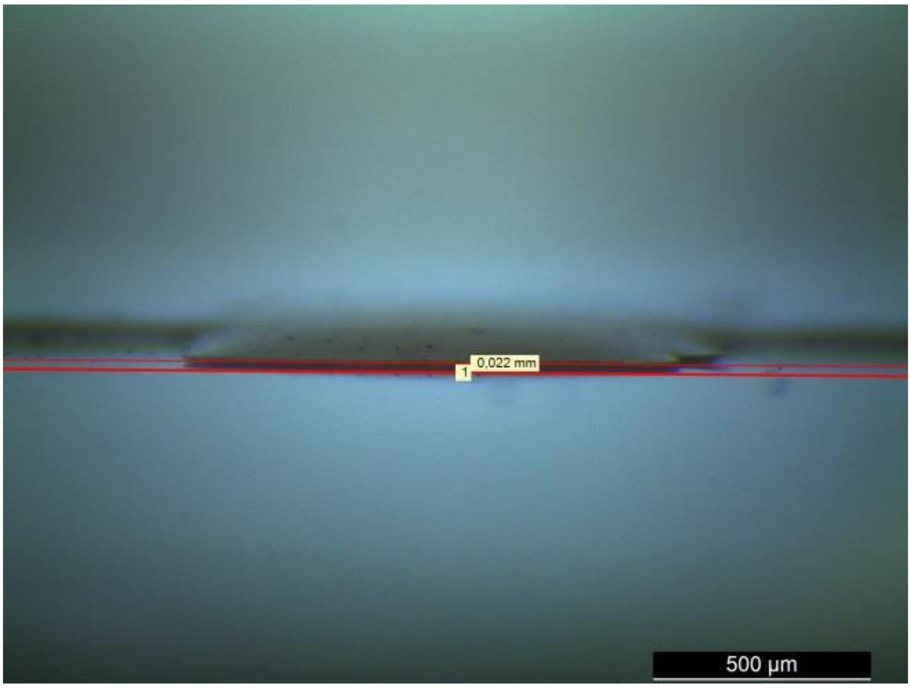

Figure S8. OM image of the air gap present at the end of the manufacturing process in a ANS device

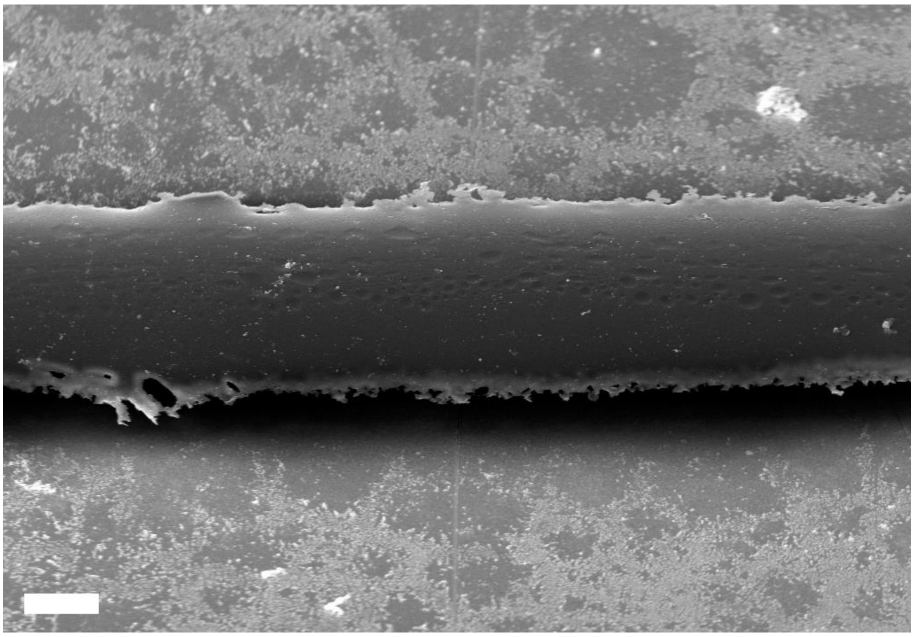

Figure S9. SEM image of one of the two springs in a ANS device

Figure S10 shows the modified Michelson interferometer used to characterize the devices. Figure S11 depicts the 3D model used to FEM simulate the behavior of the realistic devices.

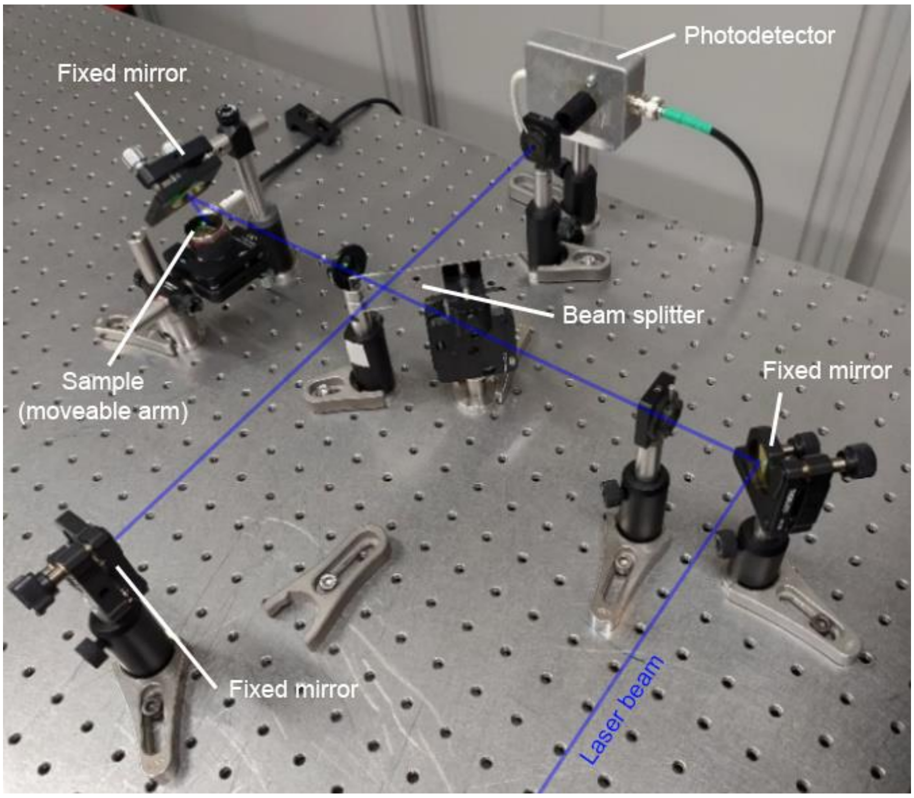

Figure S10. Modified Michelson interferometer

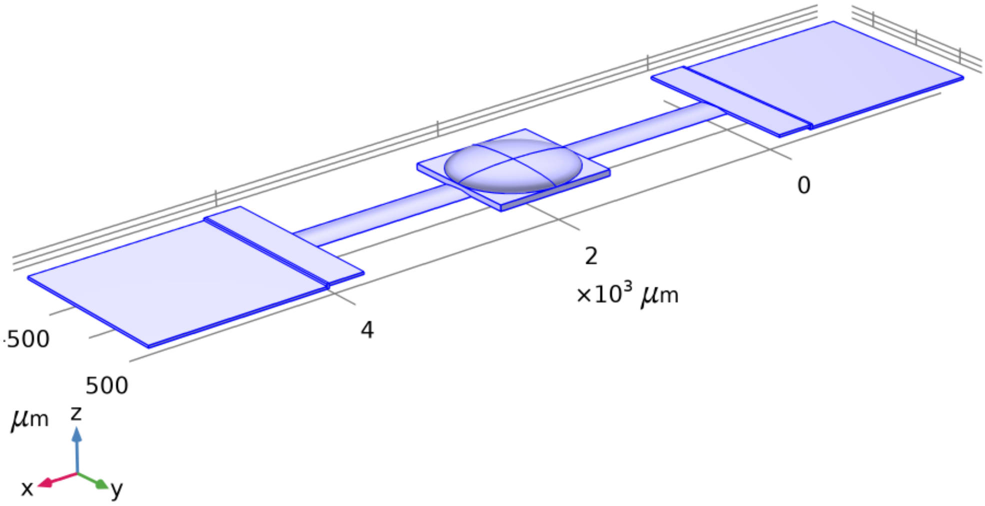

Figure S11. Realistic 3D model of a printed accelerometer

Figures S12 and S13 are representative of the displacement vs. acceleration characterization of a ANS device in LV conditions.

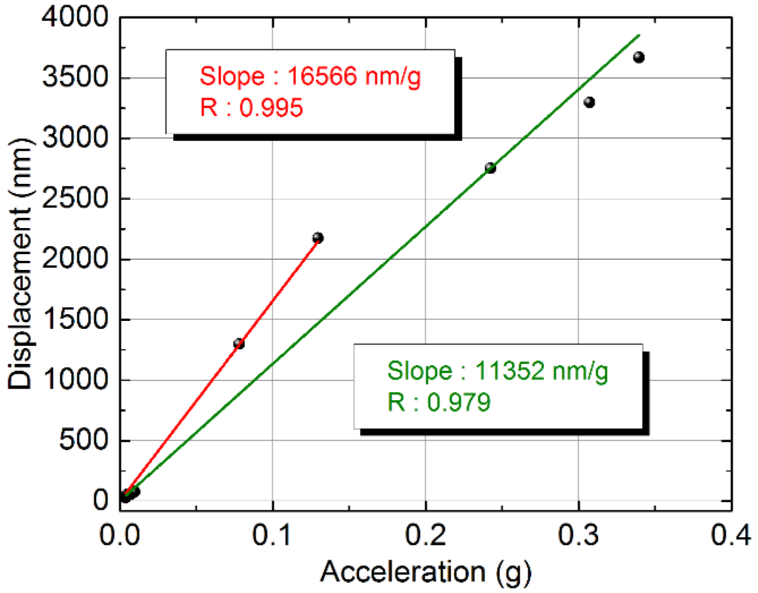

Figure S12. Displacement of the accelerometer ANS to various accelerations in LV regime

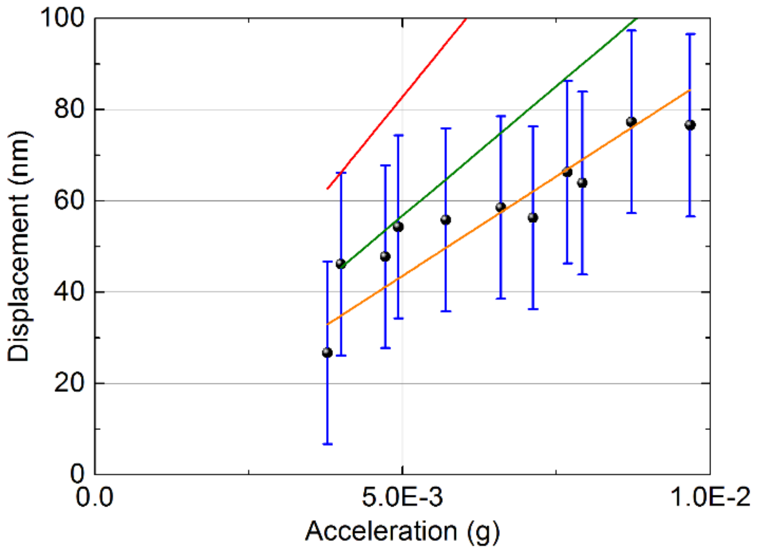

Figure S13. Fitting of the displacement vs. acceleration behavior in LV regime

Figure S14 is representative of the displacement vs. acceleration characterization of a AWS device in LV conditions.

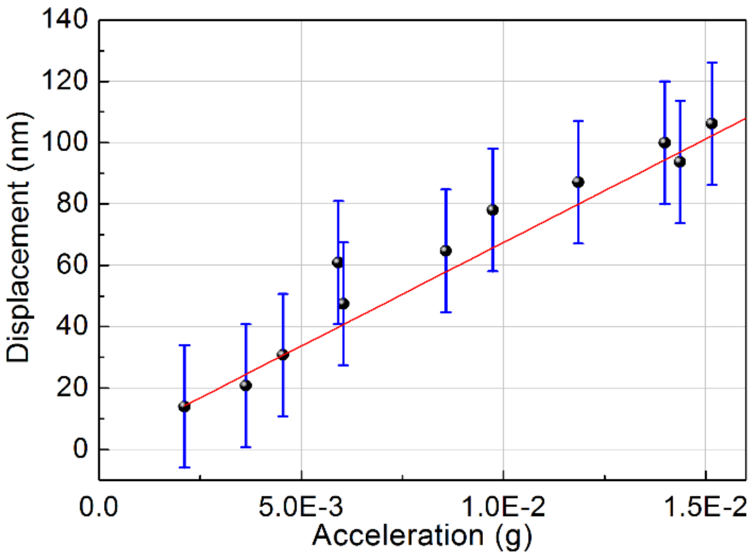

Figure S14. Displacement of the accelerometer AWS to various accelerations in LV regime
